# Comments on Boelaert et al. Determination of Asymmetric and Symmetric Dimethylarginine in Serum from Patients with Chronic Kidney Disease: UPLC–MS/MS versus ELISA. *Toxins* 2016, *8*, 149

**DOI:** 10.3390/toxins8110311

**Published:** 2016-10-27

**Authors:** Dimitrios Tsikas, Bibiana Beckmann, Pedro Araujo

**Affiliations:** 1 Center of Pharmacology and Toxicology, Hannover Medical School, Carl-Neuberg-Str., Hannover 30625, Germany; beckmann.bibiana@mh-hannover.de; 2 National Institute of Nutrition and Seafood Research (NIFES), Nordnes, Bergen N-5817, Norway; pedro.araujo@nifes.no

Boelaert et al. [1] compared UPLC–MS/MS with ELISA assays for the determination of asymmetric dimethylarginine (ADMA) and symmetric dimethylarginine (SDMA), two endogenous inhibitors of nitric oxide (NO) synthesis [2], in serum of patients with chronic kidney disease (CKD) and healthy subjects. We wish to comment on two issues arising from the paper by Boelaert et al. [1]: (1) the comparison of the methods used by these authors; and (2) the reported low protein binding (PB) of ADMA to serum proteins of healthy and CKD humans.

## 1. Method comparison of UPLC–MS/MS with ELISA

These methods were compared by linear regression analysis and by the Bland-Altman approach. From an analytical point of view, the correlation coefficients of only 0.78 for ADMA and 0.72 for SDMA are too small. The data shown in Figure 3 of the article [1] seem not to fulfil the criteria for linearity [3]. The Bland-Altman plots in Figure 3 of the article by Boelaert et al. [1] reveal a considerable disagreement between the two methods. Thus, the standard deviation of the bias is of the same order of magnitude as the actual ADMA and SDMA concentrations measured in the serum samples. The poor agreement between UPLC–MS/MS and ELISA methods [1] is consistent with the many published studies of comparative methods for ADMA measurements (e.g., Ref. [4]). Analytical shortcomings in both methods used by Boelaert et al. [1] may be responsible for the remarkable discrepancy between the estimated concentrations by UPLC–MS/MS and those by ELISA, especially in the middle concentration range (Figure 3). The fragmentation pattern of ADMA and SDMA butyl ester derivatives are different and might add to potential inconsistencies when using d_7_-ADMA as internal standard for both compounds [5]. Moreover, the matrix used for calibration might be an additional confounder in the measurement of ADMA and SDMA. Tracing a horizontal line in Figure 3 at a concentration of 2 µM SDMA for the ELISA procedure allows one to estimate a concentration range of about 0.8 µM to 3.8 µM for SDMA by the UPLC–MS/MS procedure. The estimated wide concentration range for the UPLC–MS/MS procedure undermines the credibility of the proposed agreement between UPLC–MS/MS and ELISA.

## 2. Protein binding of ADMA and SDMA

**Figure 1:**
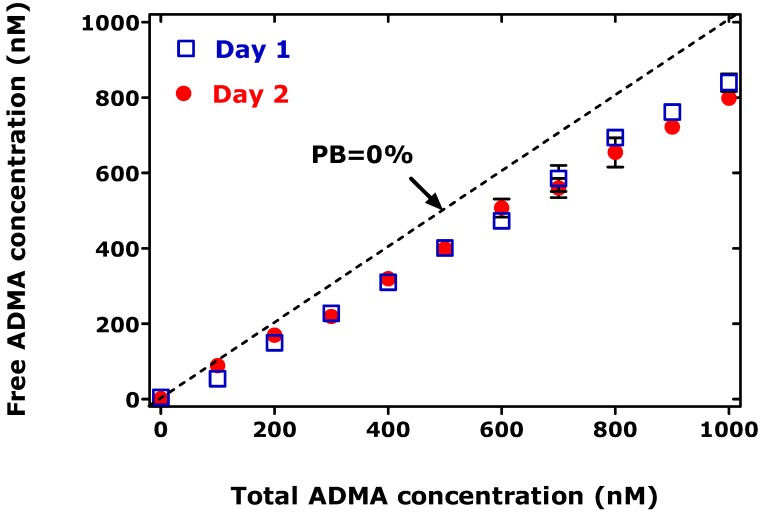
Protein binding of ADMA (0–1000 nM) to human serum albumin (60 g/L) in 67 mM phosphate buffer, pH 7.4, determined on two consecutive days. Data are shown as mean ± standard deviation from two experiments each. *y* = 1.53 + 0.80*x* (*r*^2^ = 0.998, *F* = 4542, *P* < 0.0001) for day 1; and *y* = −24 + 0.87*x* (*r*^2^ = 0.9971, *F* = 3138, *P* < 0.0001) for day 2.

The determination of the PB of drugs and endogenous substances can be performed by several methods. A crucial step in the widely used ultrafiltration technique applied by us [6] and by Boelaert et al. [1] is the use of low centrifugation forces in order not to disturb the equilibrium between the drugs or endogenous compounds and the proteins [7]. Boelaert et al. [1] used the correct formula to determine the PB of ADMA and SDMA. Regrettably, however, the PB protocol and the method used to determine the serum concentrations of ADMA and SDMA in the CKD patients and healthy controls (i.e., UPLC–MS/MS or ELISA) were not reported. The authors found that the PB of ADMA to the serum proteins of their CKD patients and healthy controls was of the order of 4% to 6.5%. Previously, we reported that the PB of ADMA to human serum albumin (HSA) in phosphate buffer (pH 7.4), as determined by GC-MS/MS using the ultrafiltration technique (300× *g* for free ADMA), ranges between about 15% and 35% and is dependent in part upon the concentration of ADMA and HSA [5] (see also Figure 1). By using the same technique we also determined mean PB values for paracetamol (acetaminophen) and salicylic acid (each at 50 µM concentration) of 27% and 97%, which are very close to the PB values reported in the literature (discussed in Ref. [6]), underlining the appropriateness of our procedure for determining the PB of ADMA to HSA. The considerably lower PB values for ADMA reported by Boelaert et al. [1] are presumably due to the use of centrifugation forces that may have been too high (but unfortunately were not reported) to generate ultrafiltrate samples for further analysis, and/or the unreliability of the analytical approach used to measure ADMA in the PB experiments. However, we cannot totally exclude the possibility that the PB value of ADMA to human serum proteins differs from those determined by us in HSA-containing phosphate buffer [6]. Therefore, we recommend that the determination of the PB of endogenous compounds such as ADMA and SDMA be carried out in buffered solutions of HSA or other relevant transport proteins in the blood. The PB of ADMA to HSA seems to be complex and warrants further mechanistic studies.
